# A validation study of the 30-day questionnaire in the national Norwegian Tonsil Surgery Register: can we trust the data reported by the patients?

**DOI:** 10.1007/s00405-023-08306-0

**Published:** 2023-11-01

**Authors:** Siri Wennberg, Marit Furre Amundsen, Vegard Bugten

**Affiliations:** 1 Department of Neuromedicine and Movement Science, Faculty of Medicine and Health Sciences, Norwegian University of Science and Technology (NTNU), 7491 Trondheim, Norway; 2 Department of Medical Quality Registries, St. Olav’s University Hospital, Torgarden, P. O. Box 3250, 7006 Trondheim, Norway; 3 Department of Otorhinolaryngology, Head and Neck Surgery, St. Olav’s University Hospital, P. O. Box 3250, 7006 Trondheim, Norway

**Keywords:** Tonsillectomy, Tonsillotomi, Medical quality register, Patient Reported Outcome Measures, Data quality

## Abstract

**Purpose:**

The aim of this study was to validate the Patient Reported Outcome Measure (PROM) in the Norwegian Tonsil Surgery Register (NTSR) and to examine whether any improvements to the questionnaire could be useful.

**Methods:**

This is a prospective, descriptive study. NTSR collects data from patients who undergo tonsil surgery and the intention of the register is to improve the quality of treatment and to contribute to research. The patients answers questions about admission due to postoperative haemorrhage, infection and pain 30 days after surgery. 305 patients were contacted on phone 1–2 weeks after answering the questionnaires electronically (ePROM) and asked the same questions. 180 of 305 patients we contacted had some kind of complications after surgery. They were asked additional questions to search for possible points for improvement of the questionnaire.

**Results:**

When comparing the results on the ePROM with the answers on phone, we found that 12 out of 14 variables achieve *almost perfect* agreement (AC_1_ ≥ 0.81). Two variables are categorized to be substantial agreement (AC_1_ = 0.61–0.80). The additional questions showed us that the questionnaire can be improved with more detailed information regarding the severity of the postoperative haemorrhage and the need of better treatment against postoperative pain.

**Conclusion:**

This study shows that the information from the 30-day ePROM has high reliability. The questions were understood as they were intended, and the answers reflect what the patients had of complications. Some changes can be done to improve the questionnaire and to open up for more research around the tonsillectomy procedure.

## Background

Medical quality registers can be an important tool for quality improvement in health care, as well as a source of data for disease monitoring and clinical or epidemiological research. Medical registers are defined as a systematic collection of clearly defined set of health and demographic data for patients with specific health characteristics, held in a central database for a predefined purpose [1]. A register is designed to address multiple questions of interest and can measure and compare results over time and between participating users. It can also be used to measure results of specific quality improvement projects [2]. National quality registers are unique tools for follow-up and result assessment [3]. In Norway, national medical quality registers are recommended to include patient-reported data [4]. In 2017, the Norwegian quality register for tonsil surgery (NTSR) was established. NTSR has the same structure and variables as the National Tonsil Surgery Register in Sweden, established in 1997 [5–8].

All national medical registers in Norway send annual reports to an executive committee who give feedback on their performance. This is an important part of the quality assurance of the registers. In the annual report, all registers have been required to specify results from Patient Reported Outcome Measure (PROM) data and how data from the register can be used for health care quality improvement [4]. PROM is in clinical research defined as reporting on the state of health directly from the patient, without interpretation by others [9]. There are two main types of PROMs that are distinguished by different levels of focus, generic, and disease-specific. In NTSR disease-specific PROM are used, with focus on specific symptoms and complications [10]. In recent years, there has been an increasing use of patient-reported data in studies to make or support decisions about individuals, groups and populations [6, 11, 12]. Patient reporting is useful for measuring the patient's own experience of treatment. It differs from traditional observed side effects, where clinicians report type and severity [13]. Patients are experts in their own health and they are important contributors in obtaining information in connection with their health care.

In order for a quality register to be used for quality improvement and research [2], as well as to have sufficient credibility in the clinical environments, the quality of the data must be high and free from measurement error [4, 14, 15]. There is an increasing demand from patients, health care providers, and payers for tools to improve quality of care and sources to increase knowledge [16, 17].

Approximately 8.000 tonsil surgery procedures are performed every year in Norway, with considerable differences in clinical practices and outcomes throughout the country. The register contains variables reported by the surgeons from the surgery and by the patients or their caregivers postoperatively. Thirty percent of the patient are children beyond 16 years [18]. The degree of completion at institution level for NTSR is high (89%) and about 80% of the patient included in the register answer ePROM [18].The register can be used to monitor clinical practices in Norway as well as monitor the implementation of new techniques in the treatment of patients with tonsil diseases [19]. The variables reported by the surgeon was validated in 2017 and the study showed that the reliability of the NTSR is high for all variables registered by the professionals at the hospital immediately after surgery [20]. Lundström et al. published in 2022 a validation study with data from the Swedish Tonsil Surgery Register where they compared register data with data in medical records [21]. Some answers in the NTSR cannot be found in the medical records, so it is important to get complete information from the patient/caregiver.

The ePROM from 30 days after surgery is a questionnaire which contains questions about complications such as haemorrhage, pain, and infection [22].

The primary aim of this study was to validate the PROM in NTSR by comparison of answers in ePROM with answers on phone, and to investigate how good the patients had understood the questionnaire. Other aims was to examine whether any improvements to the questionnaire could be useful and to identify reasons for unanswered ePROM.

## Methods

The study was conducted as a prospective study. The data quality dimensions examined in the study are reliability and relevance, as defined by Centre for Clinical Documentation and Evaluation (SKDE) [23].

In the study, all the variables used in the 30-day questionnaire were examined, together with a selection of additional questions (Table 1). Do the patients/caregivers understand the content of the questionnaires as they are intended to be understood? To investigate this, it was necessary to speak directly with the patients/caregivers themselves.

**Table 1:** Variables used in the study

**Part one**^a^
Have you contacted the health care system because of bleeding from the throat?
If Yes, how many days after the surgery did the bleeding occur?
Did the bleeding require admission to the hospital?
If yes, which hospital were you/the child admitted to?
Was another surgery performed due to bleeding?
Did any infection occur during hospital stay or within 30 days after the surgery?
If yes, what kind of infection?
Have you/the child contacted the health care system due to the infection?
Have you/the child received antibiotic treatment due to the infection?
Have you contacted health care system due to pain after the operation?
How many days after the operation did you/the child take analgesic medicine?
How many days after the operation did you/the child start eating regular food?
Did the information you received before the procedure match how the surgery and the time after was experienced?
Have you read patient information at www.halsmandeloperasjoner.no?
**Part two**^b^
Contacted the health care system due to bleeding
Who did you contact?
Which treatment was performed due to bleeding?
Contacted the health care system due to pain
Who did you contact?
The reason why patients/caregivers contact the health system due to pain
Contacted the health care system due to infection
Who did you contact?
**Part three**^c^
Have you registered electronic questionnaire that you have received?
Was the electronic form you have received too anonymous?
What would it take for you to have answered the PROM?
Technical problems to answer the questionnaire?

^a^Variables in the 30-day questionnaire

^b^Additional questions to those who had reported complications

^c^Additional questions to those who had not answered the electronic questionnaire

The data collection started in January 2020, and was completed in June the same year. The study was divided into three parts. Part one and two of the study were conducted with the same sample group. Part one was carried out by comparing data in the register with answers to the same questions given by the patients/caregivers on phone in 1–2 weeks after completing the ePROM. Part two consisted of additional questions to get more information from those who had reported in the ePROM complications after surgery. In part three, the participants who previously did not answer the ePROM received the questionnaire on paper by mail. This group also got some additional questions about why they not had answered.

The answers given by patients/caregivers on phone were written on a paper form, and then entered into Excel for analysis. The answers given on phone were compared with the original registrations in the NTSR given on ePROM.

### Data collection

The data collection was conducted during the year 2020 [24]. Two register employees (MA, SW) carried out the collection, where one (MA) had the main responsibility for the interview on phone. The call were performed 1–2 weeks after they had completed the ePROM, equally every time, and it was used a written template for the conversation. We contacted 597 patients who had received tonsil surgery and answered the electronic 30-day questionnaire, 51% (305/597) answered. When asking the questions by phone, the questions were read as similar as possible, in the same order and the same wording. The register employees who made the phone calls were blinded to the answers given on ePROM. For children under 16 years of age, the answers were given by caregivers, both ePROM and call.

In part two of the study, patients (180/305) who had one or more complications postoperatively of interest for the study were asked additional questions concerning the specific complication.

**Fig. 1:**
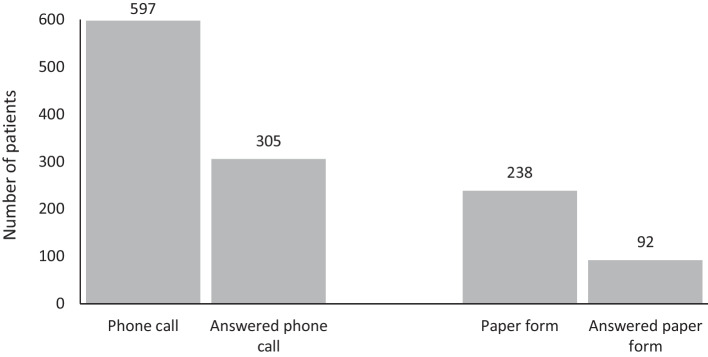
The figure show how many patients we tried to contact on phone and by paper form. The patients contacted on phone had already answered ePROM, but the patients we contacted with paper form had not answered the ePROM

In part three, 238 participants who did not answer ePROM received a 30-day PROM on paper, and additional questions about why they did not answered the ePROM. We received answer from 92 of these patients (Fig. 1).

### Statistical analyses

The results are presented by descriptive statistics and the intra-rater agreement is presented in terms of observed agreement, Cohen’s kappa and Gwet’s AC_1_ coefficients with 95% confidence intervals. Gwet's AC_1_ often shows a higher and more stable intra-rater reliability (IRR—reproducibility) coefficient than Cohen's kappa, which is previously often used in such studies. When the ratings are either negative or positive, the unbalanced prevalence of the trait will lead to an artificially reduced kappa coefficient. This will influence the kappa statistic and lead to an artificially reduced kappa coefficient [25, 26]. The AC_1_ coefficient is not affected by unbalanced trait prevalence [27, 28]. In general, AC_1_ is better suited to examine this type of compliance. In the cases included in this study with discrepancies between the kappa and AC_1_ coefficients, the reliability was considered based on the AC_1_ coefficient and the observed agreement. The Kappa and AC_1_ coefficients were interpreted as follows: ≤ 0.20, slight agreement; 0.21–0.40, fair agreement; 0.41–0.60, moderate agreement; 0.61–0.80, substantial agreement; and ≥ 0.81, almost perfect agreement.

In part three, we additionally used hypothesis test with binomial distribution to check whether there is a statistically significant difference in the proportion with complications that correspond to letters compared to those with complications that respond electronically.

The statistical analyses were computed using R statistical software.

### Patient and public involvement

The registry steering committee including a patient representative was involved in the design of the study.

## Results

### Results part one

In part one of the study, we assessed the intra-rater reliability of the 14 variables in the 30-day questionnaire in NTSR (Table 2). We made calls to 597 patient, of those 61% were female, and 27% were under 16 years. Of 597 patients/caregivers, 51% (*n* = 305) answered. In the sample of 305 who answered, 58% were female and 29% were caregivers. In this study, the caregivers who answered the call where all the same that had answered the ePROM, with an AC_1_ value of 1.

**Table 2:** Analyses of agreement

	*n*	Obs.agr.^a^ (95% CI)	Kappa (95% CI)	AC_1_ (95% CI)
Have you contacted the health care system because of bleeding from the throat?	305	0.99 (0.98–1)	0.98 (0.95–1)	0.99 (0.98–1)
If Yes, how many days after the surgery did the bleeding occur?	56	0.86 (0.79–0.95)	0.83 (0.70–0.95)	0.88 (0.74–0.95)
Did the bleeding require admission to the hospital?	56	0.98 (0.95–1)	0.96 (0.88–1)	0.98 (0.90–1)
If yes, which hospital were you/the child admitted to?	36	1.00 (1–1)	1.00 (1–1)	1.00 (1–1)
Was another surgery performed due to bleeding?	53	0.92 (0.85–0.98)	0.82 (0.65–0.99)	0.92 (0.74–1)
Did any infection occur during the hospital stay or within 30 days after the surgery?	305	0.99 (0.98–1)	0.96 (0.92–1)	0.99 (0.97–1)
If yes, what kind of infection?	44	0.89 (0.77–0.98)	0.72 (0.49–0.94)	0.89 (0.74–0.98)
Have you/ the child contacted the health care system due to the infection?	305	0.98 (0.97–1)	0.92 (0.85–0.99)	0.98 (0.97–1)
Have you/received antibiotic treatment due to the infection?	304^b^	0.99 (0.98–1)	0.95 (0.89–1)	0.99 (0.97–1)
Have you contacted health care system due to pain after the operation?	305	0.93 (0.90–0.96)	0.85 (0.79–0.91)	0.93 (0.82–0.92)
How many days after the surgery did you/the child take analgesic medicine?	304^b^	0.70 (0.65–0.75)	0.62 (0.55–0.68)	0.70 (0.59–0.71)
How many days after the operation did you/the child start eating regular food?	305	0.70 (0.64–0.75)	0.62 (0.55–0.68)	0.70 (0.59–0.70)
Did the information you received before the procedure match how the operation and the time after was experienced?	305	0.91 (0.88–0.94)	0.55 (0.39–0.70)	0.91 (0.85–0.93)
Have you read patient information at http://www.halsmandeloperasjoner.no?	304^b^	0.84 (0.80–0.88)	0.44 (0.31–0.56)	0.84 (0.71–0.84)

^a^Obs.agr. = observed agreement

^b^Missing data from one patient

Table 2 shows a high agreement between collected data by telephone and answers to the ePROM in the register. All variables showed high observed agreement ranging from 0.70 to 1.00.The values of AC_1_ showed that 12 out of 14 variables are considered to have an *almost perfect agreement*. Two out of fourteen variables fall into the category of *substantial agreement*. The kappa values showed a greater variability from 0.44 to 1.00, as expected due to the skewed trait.

### Result part two

In this part of the study, those who had reported complications got additional questions. Results showed that 56 reported that they contacted the health care system due to postoperative haemorrhage after tonsil surgery. Out of these, 64% (36 of 56) were admitted to the hospital. For the patients who were admitted after they had contacted the health care system due to bleeding, just 33% (12 of 36) stayed for observation. The rest of the patients were treated in local anesthesia (44%) or general anesthesia (23%).

For patients (*n* = 48) who contacted the healthcare system due to postoperative infection, it was of great variation who they contacted (Table 3).

**Table 3:** Contact with the health care system due postoperative complications

	Due to bleeding (*n* = 56)	Due to infectio*n* (*n* = 48)	Due to pain (*n* = 117)
Emergency Medical Communication Centre	66%	2%	–
Intermunicipal Emergency Primary Care Centre	5%	26%	20%
The surgical unit	27%	34%	55%
General practitioner	–	28%	23%
Other	2%	10%	2%

For more information, we chose to categorize the answers in four group: about why patients/caregivers contacted the health care system due to postoperative pain (*n* = 117) *Need a greater number of painkillers* (27%), *Need stronger painkillers* (34%), *Did not tolerate painkillers that were prescribed* (16%), and *Other reasons* as lack of information (22%).

### Result part three

We received responses from 39% (92 of 238 patients/caregivers) of those who answered the paper form; out of this, 60% (55 of 92) were caregivers. In the total group, 26% of the patients (24 of 92) stated that they had complications after tonsil surgery.

There were different reasons why the patients did not answer the ePROM. Technical problems related to answering the electronic questionnaire were reported from 20% of the patients/caregivers (18 of 92). It was 34% who reported that the electronic form was too anonymous, so they did not understand that they had received an ePROM. More than half of them who answered the paper form reported that they did not understand that they had received an ePROM. In this group, 43% (40 of 92) said that they would have answered the questionnaire if they had received it as a paper form.

Regarding complication rate, we found no significant difference (*p* = 0.3), neither in the group of children or for adults, between those who answered on paper forms compared to those who answered on ePROM [24].

## Discussion

Our study investigates intra-rater reliability of a PROM used in a national medical quality register. Part one in this study shows that most of the variables in the 30-day questionnaire had almost perfect reliability based on the AC_1_ values (Table 1). The questions was understood as they were intended, and the answers reflect what the patients had of complications.

We included 305 patients from the national database in the period January–June 2020. Frost et al. suggest a minimum of 200 cases for psychometric analyses in this kind of study. In some situations, a smaller sample size might also be sufficient [15]. The Goodness-Of-Fit procedure by Donner and Eliasziw states that when testing for statistical differences between moderate (0.40) and almost perfect (0.90) values, sample size estimates ranging from 13 to 66 are required [29]. Our sample of 305 patients exceeds the requisite numbers to detect generalizable estimates of intra-rater reliability.

There is an increased demand from patients to get involved in their own health care. The assessment of outcomes based on the patient's perspective using PROM are increasingly accompanying the traditional clinical ways of measuring health and the effects of treatment on the patient [30].

When evaluating the quality of surgical care, it is important that data you use are of high quality. To draw correct conclusions from a quality register, the data must be as correct as possible, with high reliability. Validation of data makes it possible to identify potential issues in one or more variables [1, 31]. It is a common opinion that the medical record is the best source for information about the patient. In the absence of “gold standard”, which the medical record often is defined as, information directly from patient/caregiver is of great value [15]. The ePROM in NTSR include some information from the patients that is not registered in the medical record. Thus, to validate all the variables in the 30-day questionnaire against the medical record is not possible. Because of this, we choose to make a personal interview with the patients/caregivers instead, as the safest way to true information.

In part two of the study, we wanted to investigate how it was possible to improve the 30-days questionnaire in NTSR. Results from part two showed that validation of data obtained by a phone interview against answers on paper or in electronic forms make it possible to identify whether there are problems with one or more questions. If a question is systematically misinterpreted or omitted to be answered, on phone, the interviewer have the opportunity to clarify the information about how the questions are intended to be answered.

The two variables with lower degree of AC_1_ value (0.7 substantial agreement) were the questions about number of days after surgery the patient took painkillers and number of days after surgery the patient got back to their ordinary food. The answer from the patients/caregivers indicated that the question about *ordinary food* was difficult to answer. What is ordinary food? An improvement of the questionnaire would be to use explanatory text for each question. The other problem with this variables was that the patient/caregivers receives the questionnaire 30 days after the procedure, and it can be difficult to remember exact number of days for use of painkillers and the number of days before consuming ordinary food. When answering questions about "Number of days after the operation they took painkillers" and "Number of days after the operation they started with normal food", it is only possible to enter one exact number (not a period like 3–5 days). These questions in the form could with advantage have been categorized with intervals. Other ways of helping the patients answering these questions right could be handing out a temporary diary describing the first 14 days after surgery. This could be used to help the patients answering the 30-days questionnaire.

The electronic questionnaire contains questions that use the term health care system. Which health care system they contacted is not possible to find in NTSR. Our study show that the patients contacted different parts of the health care system depending of what kind of trouble/complications they had. Our study showed that most of patients with postoperative haemorrhage took direct contact with Emergency Medical Communication Centre (66%) or the surgical unit (22%). For pain and infections, there were more variations regarding which part of the health care system they contacted (Table 3). This could have been specified using an alternative drop list. With a drop list, we would probably receive better information about the severity of the complication.

Results from NTSR’s annual report 2021 show that there were 22% of the patients who needed contact due to pain after the tonsil surgery [18]. According to our study, there were different reasons why the patients contacted the health care system after the tonsil surgery due to pain. The answers given by patients/caregivers on phone were categorized into four defined main groups: sufficient number of painkillers, needed stronger painkillers, did not tolerate painkillers that were prescribed, and other reasons. There are many factors that affect how the individual experiences the time after the surgery. As of today, there are no national guidelines for pain relief after tonsil surgery in Norway. If we want to use the ePROM for quality improvement of patient treatment, more detailed information about pain treatment is important. This can also be improved with a drop list.

In both the register and in the study, we found that not everyone who takes contact with the health care system due to postoperative haemorrhage are admitted to the hospital. In part two of the study, 33% of them who were admitted due to bleeding were in the hospital only for observation. Out of those who got treatment to stop the haemorrhage, 23% were treated in general anesthesia and 44% underwent treatment in local anesthesia. The way the questions in the 30-days questionnaire are formulated provides little detailed information about the severity of the haemorrhage. Regarding this complication, more detailed questions about the treatment in ePROM are needed.

In part three of the study, we wanted to investigate why the patients did not answer the ePROM, and to evaluate if the patients that did not answer had the same complication rate as those who answered ePROM. Out of 238 patients who received the paper questionnaire, there were ninety-two who answered. The reasons why these patients did not answer ePROM were complex. Some of the participants explained that the sender was too anonymous (12%), and 43% (40/92) patients/caregivers in this group answered that they would have answered if they got the questionnaire on paper. In Sweden, they use a combination of paper and electronic questionnaire which give a response rate of approximately 50% [21]. For NTSR, the technical solution today gives an 80% response rate, so to use a paper form to collect PROM-data does not seem to be a good alternative for our register [18].

In part three, the complication rate among the patients who answered the paper form and those who answered the ePROM were compared, and the result show no difference. Therefore, it seems that the complication rate is equal in the group of patients that answer the ePROM and those who answer the paper form [24]. In our study, we have a relatively small population, but studies in Sweden with larger population have found the same result [7, 21].

### Strengths and limitations

All the questions in the 30-day questionnaire are mandatory, so the completeness in NTSR is high with no missing data from the patients that answer ePROM. A standardized questionnaire was made for the telephone interview. In part one, everyone got the same questions*,* with the same word and in the same order as in the electronic 30-day questionnaire. The additional questions in part two were asked after they had answered the ePROM. The participants got the call about 1–2 weeks after they had answered the ePROM. The caregivers we spoke with on phone were all the same as the one who had answered the electronic questionnaire. Usually, we made the phone call during work/school day, and this may be one of the reasons why not everyone was able to answer the call. Those who answered the phone were all willing to answer the questions.

Choosing the optimal interval for test–retest reliability is difficult [15]. When we want to see if patients have understood the questions, it is important that their answer is not just a memory of what they have answered on ePROM, but an answer of the real situations after surgery. It would have been a strength for the study to compare the information in the medical record, as they did in Sweden [21], with the answers we received directly from the patient/caregiver. Anyway, we think our study and the Swedish study complement each other.

With additional questions in this study about the current complications, we were able to confirm their complications, even though we had not controlled the information in the medical record. Since there are no national medical system in Norway, and the patients seek different parts of the health care system, it is necessary to use the patient as a source for validation.

## Conclusion

The use of PROM has the potential to help improve the health care system and patient treatment. For this reason, high data quality is important. Our study shows that the quality of data in ePROM for NTSR has high reliability. Twelve out of fourteen questions in the ePROM are considered to have almost perfect agreement. Two questions are assessed in the analysis for substantial agreement. The study showed that it would have been an advantage to use more detailed questions to obtain more comprehensive information from the patients. Additionally, it can be useful to categorize some of the answers, use explanatory text and add drop list with alternative answers in connection to some of the questions. For a register as NTSR, the use of an electronic questionnaire seems to be the best way to collect data from the patient.

## Data sharing

The data that support the findings of this study are available from The Norwegian Tonsil Surgery Register, but restrictions apply to the availability of these data. Data from the Norwegian Tonsil Surgery Register are available upon request by researchers, but cannot be shared by the authors due to limitations in the consent given by the patients upon registration in the register. Contact Norwegian Tonsil Surgery Register by siri.wennberg@stolav.no if any request about the data from this study.

## Data Availability

Not applicable.
